# Effect of sour tea (*Lipicom*) pill versus captopril on the treatment of hypertension

**DOI:** 10.12861/jrip.2015.15

**Published:** 2015-09-01

**Authors:** Ali-Reza Soleimani, Hossein Akbari, Saeid Soleimani, Seyed Seifollah Beladi Mousavi, Mohamad-Reza Tamadon

**Affiliations:** ^1^Department of Internal Diseases, Kashan University of Medical Sciences, Kashan, Iran; ^2^Department of Statistics, Kashan University of Medical Sciences, Kashan, Iran; ^3^Department of Internal Diseases, Kashan University of Medical Sciences, Kashan, Iran; ^4^Chronic Renal Failure Research Center, Ahvaz Junishapur University of Medical Sciences, Ahvaz, Iran; ^5^Department of Internal Medicine, Semnan University of Medical Sciences, Semnan, Iran

**Keywords:** Hypertension, Sour tea, Captopril

## Abstract

**Introduction:** Herbal medicines are traditionally prescribed to manage blood pressure.

**Objectives:** We aimed to evaluate effect of sour tea pill containing the herb’s extract versus captopril on the treatment of hypertension.

**Patients and Methods:** In our crossover clinical trial 20 patients were enrolled in the study and advised for life style modification then the participants were randomly divided into 2 groups. Sour tea pills was prescribed at a dose of 500 mg and captopril at a dose of 12.5 mg twice daily. In order to improve precision and final measurement, ambulatory blood pressure monitoring (ABPM) was performed both prior and after measuring the hypertension in 2 successive visits. After 6 weeks of therapy, the methods changed and 6 weeks later ABPM was performed three times (baseline, at end of the 6th and 12th week). The 2 groups were merged together before data analysis.

**Results:** Of the 20 patients, 13 (65%) were male and 7 (35%) were female. No significant difference of sex, age, and job was detected between 2 groups (*P* ≥ 0.05). Mean decreasing in systolic blood pressure was 7.75 ± 8.3 and 13.3 ± 16.1 mm Hg in the captopril and sour tea groups, respectively. Also, mean decline in diastolic blood pressure decreases was 2.15 ± 4.14 and 5.8 ± 7.8 mm Hg for captopril and sour tea groups, respectively. No side effect was observed in the sour tea pill group in the study.

**Conclusion:** According to the effect of sour tea pill on decreasing blood pressure, without giving priority over captopril, sour tea pill containing the herb’s extract can be prescribed as an adjuvant therapy for lowering the prescribed dosage of captopril.

**Implication for health policy/practice/research/medical education:**

In this study we indicated the antihypertensive effect of sour tea, while its priority over captopril is not proved. It could be administrated as a supportive therapy to minimize the dose of the drug used.

## Introduction

Approximately one billion people worldwide are suffering from high blood pressure. Arterial pressure (hypertension) is known as the most common and main risk factor for public health in the developed countries, so that half of the population living in some communities are affected by this health problem. In more than 90% of cases, cause of the disease is unknown which is distinguishable under the title of essential or primary hypertension (1). Race, age, high cholesterol, smoking, alcoholism and obesity are among the underlying causes for the disease. Serious cardiopulmonary and renal complications are manifested if the disease is not managed duly and promptly. Most of the patients with heart complications are faced with early death (1-5). As the results obtained from the clinical trials suggest, treating hypertension can contribute to about 35%-40% decrease in heart stroke, 20%-25% decrease in myocardial infarction, and 50% decrease in heart failure (6,7).

Diuretics, beta-blockers, alpha-adrenergic inhibitors, angiotensin converting enzyme (ACE) inhibitors, vasodilators, angiotensin receptor (ARB) II blockers and calcium-channel antagonists are among the common drugs which are used for the treatment of hypertension and are associated with some complications such as headache, dizziness, heart and/or renal failure (1-8).

Herbal medicines such as olive leaves, garlic and sour tea in particular are conventionally prescribed to control blood pressure (9). Studies which have investigated the effects of herbal medicines on blood pressure prove that sour tea (*Hibiscus sabdariffa*) leaves have a remarkable effect on decreasing hypertension (10-12). The first report regarding the hypotensive effect of the drug dates back to 1936 (13). Effectiveness of the herb is mostly attributable to flavonoids, while its hypotensive effect is due to its diuretic characteristic and its role in inhibiting ACE.

## Objectives

Due to the chronic nature of the disease and adverse effects of the drugs available, herbal medicines can be prescribed exclusively or as adjuvant therapies alongside the standard treatments in order to alleviate the side effects and improve quality of life of the patients with hypertension. The present study was designed to investigate the effect of sour tea pills versus captopril in the treatment of hypertension.

## Patients and Methods

**Figure 1:**
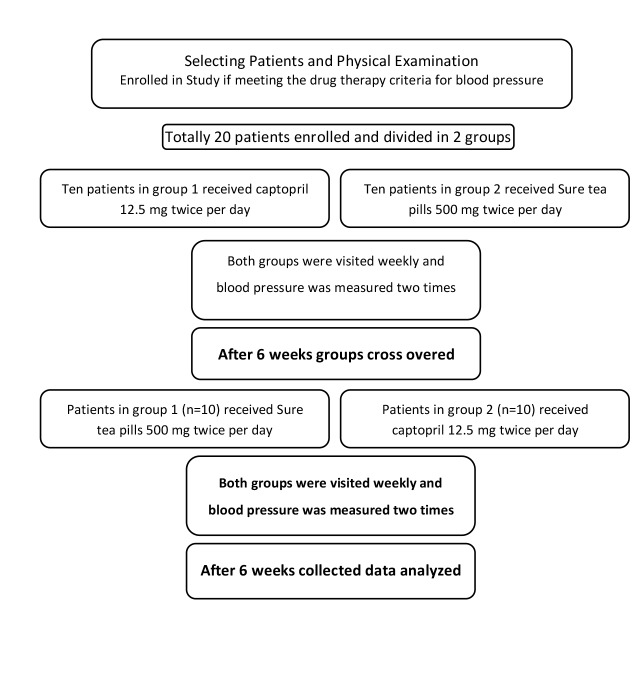
Study design diagram

This crossover clinical trial was conducted initially on 20 patients aged more than 18 years old (Figure 1). All the participants met the drug therapy criteria for blood pressure based on at least 2 measurements of mean blood pressure which were performed accurately at sitting position during at least 2 office visits. The subjects were included according to the JNC-7 classification system for hypertension and those who did not meet the drug therapy criteria were excluded from the study, however, they were advised for life style modification and other methods. The subjects were randomly divided into 2 sour tea pill and captopril pill groups which were followed up within a 6-week period. Sour tea group received a dosage of 500 mg of hyporex with ACE inhibitor effect twice a day and captopril pill group received 12.5 mg of drug twice a day. The patients were examined through 2 successive visits in a week. Their blood pressure was measured accurately for 2 times at each visit. If the blood pressure was not in its normal level (120/90 mm Hg), another alternative drug was prescribed. Each drug was given for another 6-week period. After measuring the blood pressure within 2 successive visits, ambulatory blood pressure monitoring (ABPM) was performed to improve the accuracy of results and final measurement. This procedure was followed using the modest set made in Germany. Patients’ blood pressure was examined for 24 hours (every 20 minutes during day time and every 30 minutes during sleep time). Also, the variables of hypertension dipping (a 15% decline of blood pressure during sleep time) and controlled blood pressure during day time and sleep time were recorded as 140/90 mm Hg and 125/75 mm Hg, respectively.

Mean change of blood pressure during each of the time variables were set to be less than 15%. In case of having appropriate ABPM, the patients were put in captopril group receiving a dose of 12.5 mg twice a day or sour tea group receiving a dose of 500 mg twice a day (produced by Barij Essence Co Iran, Kashan.) for a 6-week period. To study the electrolyte imbalances and possible renal or liver abnormalities, the patients were examined at 3 treatment stages (at the beginning of stage one, at the beginning of stage 2, and at the end of stage 2, respectively). In the presence of any complication at any stage, the patient was excluded from the study. The second stage was just commenced after the sixth week of treatment by the substitution of captopril for sour tea pills without washout period.

### Ethical issues

The research followed the tenets of the Declaration of Helsinki. Informed consent was obtained and the research was approved by the Ethics Committee of Kashan University of Medical Sciences.

### Data analysis

Randomization was achieved through using randomized block. Finally, the selected patients were sent to drug stores all located in the neighborhood of the offices to receive their prescriptions. Two groups were merged together before data analysis. The data were analyzed using chi-square, Fisher exact and paired *t* tests. The groups were also compared using Mann-Whitney U and *t* tests.

## Results

This study was designed to investigate the effect of sour tea on decreasing hypertension for the patients referring to nephrology and hypertension clinic of Kashan. The study included 114 patients of which 94 patients were excluded from the study (because of complications, not visiting the physician, metabolic abnormalities or history of taking antihypertensive drugs). At last, 20 patients were studied in the 2 captopril and sour tea groups. All the patients were put in mild and median hypertension categories.

As shown, 13 (65%) and 7 (35%) individuals were male and female, respectively. No significant difference was observed among the patients in terms of age (*P *> 0.05). Mean age of the patients in the captopril and sour tea groups was 46.5 ± 12 and 53.3 ± 9 years, respectively. No significant difference of age and job between groups was observed (*P *> 0.05).

No significant statistical difference of the risk factors such as smoking, diabetes and hyperlipidemia between groups was found (*P *> 0.05; Table 1).

**Table 1:** Frequency distribution of hypertensive patients in the 2 groups by demographic variables and risk factors

**Variables**	**Group**	**Captopril (%)**	**Sour tea (%)**	***P ***
Sex	Male	6 (60)	7 (70)	NS
	Female	4 40)	3 (30)	
Age	Less than 50 y	6 (60)	4 (40)	0.371
	50 y and more	4 (40)	6 (60)	
	Mean±SD	46.5 ± 12	53.3 ± 9	0.169
Job	Employee	2 (20)	4 (60)	0.84
	Unofficial	4 (40)	5 (50)	
	Housekeeper	4 (40)	1 (10)	
Smoking	Positive	7 (70)	8 (80)	NS
	Negative	3 (30)	2 (20)	
Diabetes	Positive	2 (20)	0 (0)	0.474
	Negative	8 (80)	10 (100)	
Hyperlipidemia	Positive	1 (10)	2 (20)	NS
	Negative	9 (90)	8 (80)	
LD consumption	Positive	0 (0)	1 (10)	NS
	Negative	10 (100)	9 (90)	
Familial Background	Positive	8 (80)	6 (60)	0.628
	Negative	2 (20)	4 (40)	

NS, not significant.

Table 1 shows mean body temperature was 36.95°C and 36.92°C in the captopril and sour tea groups, respectively(*P *> 0.05). Mean heart rate in the captopril and sour tea groups was 78.9 and 81.8, respectively (*P *> 0.05).

As the results showed, mean body weight was 79.9 kg and 81 kg in the captopril and sour tea groups, respectively. Mean height was 170 and 169.88 in the 2 groups, respectively. As the findings show, no significant difference of body mass index between 2 groups was detected (*P *> 0.05; Table 2).

**Table 2:** Statistical indices for vital signs and hypertension risk factors at the beginning of the study

**Variables**	**Group**	**No.**	**Average**	**SD**	***P***
Temperature	Captopril	10	36.95	0.16	0.806
	Sour tea	10	36.92	0.35	
Heart beat	Captopril	10	78.9	6.05	0.244
	Sour tea	10	81.8	4.64	
Weight	Captopril	10	79.9	9.01	0.964
	Sour tea	10	81	10.93	
Height	Captopril	10	170	10.87	0.963
	Sour tea	10	169.88	77.96	
BMI	Captopril	10	28.03	5.05	0.695
	Sour tea	10	27.03	4.36	

Abbreviation: BMI, body mass index.

Likewise mean systolic blood pressure at the beginning of the study was 153 and 154 mm Hg for the captopril and sour tea groups, respectively. Similarly, mean diastolic blood pressure was 96.9 and 98 mm Hg for 2 groups, respectively. Initially, no significant difference of blood pressure between 2 groups was observed (*P *> 0.05; Table 3).

**Table 3:** Statistical indices for essential hypertension

**Variables**	**Group**	**No.**	**Average**	**SD**	***P***
Systolic BP	Captopril	10	153	5.1	0.69
	Sour tea	10	154	5.91	
Diastolic BP	Captopril	10	96.9	3.68	0.502
	Sour tea	10	98	3.49	
Systolic BP while slept	Captopril	10	137.3	6.3	0.588
	Sour tea	10	139.3	9.57	
Diastolic BP while slept	Captopril	10	91	7.37	0.885
	Sour tea	10	91.5	7.84	
Systolic BP while awake	Captopril	10	147	6.32	0.857
	Sour tea	10	147.5	5.89	
Diastolic BP while awake	Captopril	10	98	4.83	0.82
	Sour tea	10	97.5	4.85	

Abbreviation: BP, Blood pressure.

Additionally mean systolic blood pressures at sleep time was 137.3 and 139.3 mm Hg, respectively, in the captopril and sour tea groups at the beginning of the study. In this study, no significant difference systolic and diastolic blood pressures at night time and day time between 2 groups was detected (*P *> 0.05).

The result of this study showed an initial dipping in 40% and 70% of the captopril and sour tea groups, respectively, however the difference was not significant between the 2 groups (*P *> 0.05).

Ocular problems were present among all of the patients (100%) in both groups, while evidence of cardiac disease was only found in one patient in the captopril group. In terms of ocular and electrocardiogram (EKG) abnormalities, no significant difference was found between the 2 groups (*P *> 0.05).

Mean systolic blood pressure in the captopril group was initially 152.8 mm Hg which changed to 145.01 mm Hg after the treatment (a 7.75 mm Hg decrease). Diastolic blood pressure at sleep was 134.4 mm Hg initially that became 109.8 mm Hg (a 24.6 mm Hg decrease). Mean diastolic blood pressure at the beginning was 94.7 mm Hg which changed to 92.55 mm Hg after the treatment (a 2.15 mm Hg decrease). Diastolic blood pressure at sleep time was 86.75 mm Hg at the beginning which converted to 70.5 mm Hg (a 16.25 mm Hg decrease). A significant decrease in blood pressure indices was observed after the treatment (*P* < 0.001; Table 4).

**Table 4:** Statistical comparison of blood pressure before and after treatment in the captopril group

**Blood pressure**	**Before treatment (n = 20)**	**After treatment (n = 20)**	**Changes**	***P ***
	**Mean ± SD**	**Mean ± SD**		
Systolic	152.8 ± 5.6	145.05 ± 10.7	7.75 ± 8.3	0.001
Systolic while slept	134.4 ± 7.85	109.8 ± 7.8	24.6 ± 10.8	<0.001
Systolic while awake	146 ± 4.7	122.3 ± 8.8	23.78 ± 9.01	<0.001
Diastolic	94.7 ± 4.2	92.55 ± 6.46	2.15 ± 4.14	0.032
Diastolic while slept	86.75 ± 8.3	70.5 ± 3.6	16.25 ± 8.25	<0.001
Diastolic while awake	94.8 ± 5.3	79.8 ± 4.7	15 ± 7.07	<0.001

Mean systolic blood pressure in the sour tea group was initially 153 mm Hg that was decreased to 139.7 mm Hg after the treatment (a decline of 13.40 mm Hg). Considering systolic blood pressure at sleep time, it was 138.4 mm Hg at the beginning which changed to 125.5 mm Hg (a 12.9 mm Hg decrease). Mean diastolic blood pressure was initially 94.85 mm Hg which became 89.02 mm Hg after the treatment (a decrease of 5.8 mm Hg). Diastolic blood pressure at sleep time was 90.25 mm Hg at the beginning which became 80.5 mm Hg (a 9.75 mm Hg decrease). No significant decrease was observed in blood pressure indices after the treatment (*P* < 0.001; Table 5).

**Table 5:** Statistical comparison of blood pressure before and after treatment in the sour tea group

**Blood pressure**		**Before treatment (n = 20)**	**After treatment (n = 20)**	**Changes**	***P***
		**Mean±SD**	**Mean±SD**		
Systolic		153 ± 5.5	139 ± 14.8	13.4 ± 16.1	0.004
ABPM	Systolic while slept	138.4 ± 8.12	125.5 ± 6.12	12.9 ± 2.13	<0.001
	Systolic while awake	146 ± 4.75	134.85 ± 5.9	11.15 ± 6.8	<0.001
Diastolic		94.85 ± 4.4	89.02 ± 5.2	5.8 ± 7.75	0.007
ABPM	Diastolic while slept	90.25 ± 6.2	80.5 ± 6.4	9.75 ± 10.2	<0.001
	Diastolic while awake	94.75 ± 5.25	87 ± 4.1	7.75 ± 6.38	<0.001

Abbreviation: ABPM, ambulatory blood pressure monitoring.

The decrease in mean systolic blood pressure in the captopril and sour tea groups was 7.75 and 13.3 mm Hg, respectively. Also, the decrease in mean diastolic blood pressure was 2.15 and 5.8 mm Hg in the captopril and sour tea groups, respectively. The difference between the reduction of systolic and diastolic blood pressures was not significant (*P *> 0.05) between the 2 groups. However, the decrease in systolic (*P* < 0.001) and diastolic (*P* < 0.05) blood pressure at sleep and day time was significant after the treatment (Table 6).

**Table 6:** Statistical indices for blood pressure after treatment in both groups

**Variables**		**Statistical indices**	**Before treatment (n = 20)**	**After treatment (n = 20)**	**Changes**	**P**
			**Mean ± SD**	**Mean ± SD**		
Systolic		Captopril	20	7.75	8.3	0.309
		Sour tea	20	13.3	16.1	
ABPM	Systolic slept	Captopril	20	24.6	9.2	<0.001
		Sour tea	20	12.9	9.54	
	Systolic awake	Captopril	20	23.75	9.01	<0.001
		Sour tea	20	11.15	6.8	
Diastolic		Captopril	20	2.15	4.14	0.092
		Sour tea	20	5.8	7.8	
ABPM	Diastolic slept	Captopril	20	16.25	8.25	0.033
		Sour tea	20	9.75	10.2	
	Diastolic awake	Captopril	20	15	7.1	0.002
		Sour tea	20	7.75	6.4	

As shown, 55% of the subjects in captopril group with primary dipping and 35% of the sour tea group without primary dipping revealed secondary dipping. The difference between the two dipping status was not significant in the captopril group (*P *> 0.05). While in the sour tea group, 55% of the patients with primary dipping and 30% of the patients without primary dipping showed secondary dipping. The difference between the 2 types of primary dipping and secondary dipping was significant in the sour tea group (*P* < 0.05).

Four cases in captopril group had abnormal high-density lipoprotein cholesterol (HDL-C) tests which 3 cases remained abnormal after the treatment that difference was not significant (*P *> 0.05). However, of the 4 abnormal HDL-C tests in the sour tea group, 3 cases became normal after the treatment (*P *> 0.05). Low-density lipoprotein cholesterol (LDL-C) and triglyceride tests in both groups, and total cholesterol in the captopril group showed no significant difference (*P* < 0.05; Table 7).

**Table 7:** Frequency distribution of primary dipping based on secondary dipping in both groups

	**Captopril, secondary amounts**			**Sour tea, secondary amounts**		
	**Normal**	**Abnormal**	**SC**	**Normal**	**Abnormal**	**SC**
HDL	1	3	0.004	3	1	0.2
LDL	4	5	0.011	4	6	0.11
Triglyceride	3	7	0.002	2	8	<0.001
Cholesterol	4	6	0.011	6	4	0.87
Fasting blood sugar	0	1	0.1	1	0	1

Abbreviations: HDL, high-density lipoprotein; LDL, Low-density lipoprotein; SC, serial correlation.

All the tests for blood cells count in both groups were normal before and after the treatment (Table 8).

**Table 8:** Frequency distribution of blood cells count before and after treatment in both groups

	**Captopril, secondary amounts for blood cells count**			**Sour tea, secondary amounts for blood cells count**		
	**Normal**	**Abnormal**	**SC**	**Normal**	**Abnormal**	**SC**
Normal	20	0	-	20	0	-
Abnormal	0	0	-	0	0	-

Abbreviations: SC, serial correlation.

Ten cases in sour tea group had abnormal aspartate aminotransferase (AST) which 6 cases remained abnormal after the treatment that difference was not significant (*P *> 0.05). Also, of the 10 abnormal AST cases in the captopril group, only one case was abnormal after the treatment which was not significant (*P *> 0.05). ALT changes were significant in both groups after the treatment (*P* < 0.05). Blood urea nitrogen (BUN), creatinine, and potassium test results were all normal in the two groups before and after the treatment (Table 9).

**Table 9:** Frequency distribution of the hepatorenal function indices before and after treatment in both agroups

**Primary amounts**		**Captopril**			**Sour tea**		
		**Secondary amounts**			**Secondary amount**		
		**Normal**	**Abnormal**	**SC**	**Normal**	**Abnormal**	**SC**
AST	Normal	9	1	0.057	8	2	0.17
	Abnormal	4	6	0.057	4	6	0.17
ALT	Normal	13	0	0.007	12	0	0.036
	Abnormal	3	4	0.007	4	3	0.036
BUN	Normal	20	0	-	20	0	-
	Abnormal	0	0	-	0	0	-
Creatinine	Normal	20	0	-	20	0	-
	Abnormal	0	0	-	0	0	-
Potassium	Normal	20	0	-	20	0	-
	Abnormal	0	0	-	0	0	-

Abbreviations: AST, aspartate aminotransferase; ALT, alanine aminotransferase; BUN, bood urea nitrogen.

## Discussion

This study was conducted on 20 hypertensive patients to evaluate the effect of sour tea pills versus captopril in lowering hypertension. Based on the findings, the 2 groups under the study manifested no significant difference in terms of variables such as age, sex, job, hypertensive risk factors, vital signs, body mass index, essential hypertension, ABPM statistical indices, primary dipping, ocular, cardiac problems and primary tests results. This finding proved that the 2 groups were entirely the same, and indicated that the difference in the results obtained for the 2 groups regarding the decrease in blood pressure were not attributable to the underlying variables mentioned above. Captopril and sour tea groups had a significant decrease in systolic and diastolic blood pressures both at sleep and day time. This proved that sour tea pill and captopril could significantly decrease blood pressure in the patients due to their antihypertensive effect. Also, in the newly conducted studies, the effect of sour tea in lowering blood pressure has been emphasized. Herrera-Arellano et al (10) studied the effect of sour tea in lowering mild to severe blood pressure. Haji Farajian and Haji Tarkhani (11) reported that sour tea extract could affect the essential hypertension and control it to some extent. Adegunloye et al (12) phytochemically studied the mechanism of sour tea in lowering blood pressure and reported it as a suitable medicine for controlling hypertension. Wahabi et al (14) conducted a systematic review on researches which had investigated the effect of sour tea in lowering hypertension and concluded that due to the diversity of chemical compounds in sour tea, it could potentially have various benefits among which the decrease in blood pressure is one of the most important effects. Also, McKay et al (15) pointed out the effective role of our tea in controlling prehypertensive and mild hypertensive patients. In a study carried out by Ojeda et al, (16) about the mechanisms of sour tea in lowering blood pressure, the decrease was attributed to the existence of converting enzyme (ACE) inhibitors which is present in sour tea compounds. Mozaffari-Khosravi et al (17) reported a very desirable effect of sour tea extract in controlling hypertension among type diabetic II patients.

Our findings demonstrated that sour tea was not significantly superior to captopril. However, in case of systolic blood pressure at sleep and day time, captopril seemed to have a more significant effect in decreasing hypertension. Different studies have reported the efficacy of sour tea. Wahabi et al (14) studied the antihypertensive effect of sour tea versus other standard drugs. Additionally, Herrera-Arellano et al (10) reported that sour tea was as effective as beta-blockers. Adegunloye et al, (12) in their phytochemical study investigated the mechanism of sour tea and concluded that it was as effective as converting enzyme (ACE) inhibitors. McKay et al (15) proved antihypertensive effect of sour tea in patients with mild and prehypertension, and reported that the effect of sour tea was similar to the effects of beta-blockers and calcium-channel blockers. Ojeda et al (16) studied the mechanism of sour tea in lowering blood pressure and concluded that its effect was as great as other alternative drugs because of the existence of converting enzyme (ACE) inhibitors in its compounds.

On the other hand, Haji Faraji and Haji Tarkhani (11) reported that sour tea extract was effective in treating essential hypertension, but its superiority over other standard anti-hypertensive drugs was not claimed. In the study by Mozaffari-Khosravi et al, (17) they found that sour tea extract was very effective in lowering blood pressure in the type diabetic II patients, whereas they believe that this effect was much less than that for drugs such as captopril and beta-blockers.

Initially, captopril and sour tea pills showed remarkable antihypertensive effect in the patients. However, sour tea then appeared to be less effective than captopril.

## Conclusion

This study showed the antihypertensive effect of sour tea into account, while its priority over captopril is not proved. It might be administrated as a supportive therapy to minimize the dose of the drug used.

## Conflicts of interest

The authors declare no conflicts of interest.

## Authors’ contribution

All authors contributed to the manuscript equally.

## Funding/Support

This article was extracted from a thesis by Saeid Soleimani. This study was supported by a grant from Kashan University of Medical Sciences (grant No. 55, 1389).

## Acknowledgments

The author would like to appreciate the Research and Educational Deputies of Kashan University of Medical Sciences.
